# MIMO: an efficient tool for molecular interaction maps overlap

**DOI:** 10.1186/1471-2105-14-159

**Published:** 2013-05-15

**Authors:** Pietro Di Lena, Gang Wu, Pier Luigi Martelli, Rita Casadio, Christine Nardini

**Affiliations:** 1 CAS Key Laboratory For Computational Biology Chinese Academy of Sciences - Max Plank Institute Partner Institute for Computational Biology, Yue Yang Road 320, Shanghai, 200031, P.R.C; 2 Department of Computer Science and Engineering - DISIUniversity of Bologna, Mura Anteo Zamboni 7, Bologna, 40126, Italy; 3 Biocomputing Group, University of Bologna, Via San Giacomo 9/2, Bologna, 40126, Italy

## Abstract

**Background:**

Molecular pathways represent an ensemble of interactions occurring among molecules within the cell and between cells. The identification of similarities between molecular pathways across organisms and functions has a critical role in understanding complex biological processes. For the inference of such novel information, the comparison of molecular pathways requires to account for imperfect matches (flexibility) and to efficiently handle complex network topologies. To date, these characteristics are only partially available in tools designed to compare molecular interaction maps.

**Results:**

Our approach MIMO (Molecular Interaction Maps Overlap) addresses the first problem by allowing the introduction of gaps and mismatches between query and template pathways and permits -when necessary- supervised queries incorporating *a priori* biological information. It then addresses the second issue by relying directly on the rich graph topology described in the Systems Biology Markup Language (SBML) standard, and uses multidigraphs to efficiently handle multiple queries on biological graph databases. The algorithm has been here successfully used to highlight the contact point between various human pathways in the Reactome database.

**Conclusions:**

MIMO offers a flexible and efficient graph-matching tool for comparing complex biological pathways.

## Background

In the post-genomic era the analysis of biological networks plays a crucial role in computational and systems biology. Consequently, biological network databases, tools for biological graph modeling and approaches for the management and standardization of the large amount of generated data are under continuous evolution. As an example, the PathGuide [1] repository lists four different XML standards for biological networks, modeled as graphs (SBML [2], BioPax [3], CellML [4], PSI-ML [5]), and over 300 biological pathway related resources, including databases of protein-protein interactions, metabolic and signaling pathways, gene regulatory and interaction networks. Generally speaking, in these databases, different molecular species are represented as nodes, while edges indicate a plethora of relationships existing among such molecules (including protein-protein interactions and phosphorylation).

Due to the increasing availability of biological graph databases, the problem of developing efficient and flexible *subgraph matching* methods arises in a number of applications. The main goal of biological graph matching is to detect the template-subgraphs that share similarity with noisy pathways built from the analysis of experimental data or built by collecting various sources of information. The goals are numerous and span over a large amount of topics: inference of metabolic pathways [6], prediction of protein-protein interactions (PPI) [7,8] or complex interactions by joining together various sources of information [9]. In the frame of the quickly evolving translational and evidence-based medicine it is crucial to give biological support to any novel claim, emerging from statistically relevant clinical evidence. In this sense, from the identification of shared pathways among maladies [10], to the elucidation of common targets for different drugs [11], and potential side effects, it is crucial to provide biological bases on any novel finding. Challenges involve both computational bottlenecks (such as the computational intractability of the subgraph matching problem, which translates in huge time/memory requirements), and biological limitations, due to noisy/incomplete information.

In the last ten years, several approaches to perform *approximate graph matching* have been proposed [12-22]. However, few of them are specific for biological graph comparisons. The most notable examples are Netalign [8], Rahnuma [6], PathAligner [14], PathBLAST [15], NetworkBLAST [17], and SAGA [20]. To compare PPI, Netalign [8] models pathways as undirected graphs, allowing mismatches up to a certain BLAST E-value and gaps by clustering smaller subnetworks (overlap ≥80*%*, i.e. gaps limited to a proportion of the size of the smaller network). PathBLAST [15] and its extension NetworkBLAST [17] allow node mismatches and gap-node management for very short pathways. For the modeling of metabolic network Rahnuma [6] uses directed hypergraphs, with large flexibility in terms of gaps and mismatches (backtracking). However, the approach does not rely on any standard. PathAligner [14] can only process *linear* pathways, therefore excluding tree-like structures. SAGA [20] permits both node gaps and mismatches and implements a very computationally efficient subgraph indexing procedure, which, however, affects its sensitivity (i.e a pathway of *exactly* three nodes can be indexed only if there exists a path joining each possible pair of nodes) and does not allow the management of purely linear pathways (that have no backward edges and thus are not indexed). Besides, the input is SAGA-specific. Overall, the above described approaches rely on simplified graphs topologies (for example, none of these methods can handle directed edges) and have consequently limited flexibility or reduced matching capabilities.

Our contribution, MIMO (Molecular Interaction Maps Overlap) is characterized by three main properties that guarantee the suppleness needed to properly handle the biological graph matching problem. First, our algorithm relies directly on the graph topology described in SBML documents, which defines a *reaction* as a set of interacting entities (*reactants, products of the reactions and modifiers*) placed in specific *compartments*. This takes into account the biological environment and the complexity of a 3-ways interaction, crucial to preserve the largest possible information in noisy biological network models. Importantly, no intermediate format conversion is needed, avoiding possible losses of information and additional error-prone processing steps. Second, our matching procedure naturally allows node mismatches and gap-nodes introduction. Although computationally intractable, the matching procedure implemented in MIMO is fast enough to allow multiple queries on biological graph databases. Third, in order to deal with the possibility to match distinct elements with similar biological role (i.e. orthologous proteins), or conversely not to match distinct entities encoded as the same element (i.e. a gene and its expressed protein), MIMO allows the user to specify a set of allowed/forbidden mappings between entities in two SBML documents. This list is preprocessed to define a similarity function that extends the matching capabilities of the procedure, when the user has *a priori* knowledge about the biological processes to be compared.

## Implementation

### SBML format and graph model

The Systems Biology Markup Language (SBML) [2] is a free XML-based format for representing molecular interaction networks. In the following, we only describe those components of an SBML document that are used in our comparison algorithm (for additional details refer to [23]).

An SBML document specifies a set of entities, generally denoted with the term *species*, that take part in *reactions*. An SBML species has two mandatory attributes: the *id attribute*, which uniquely identifies the species in the document, and the compartment attribute, the physical location where the reacting species are placed. A species has also an optional *name attribute* of type string. Distinct species (i.e. species with distinct id attributes) are allowed to share the same name (for example, a gene and its expressed protein). A *reaction* component is a statement that links one or more species. A reaction is defined in terms of the participating species and it consists of a set of (possibly empty) *reactants*, *products* and *modifiers*, along with an additional Boolean *reversibility attribute*. Just like species, reactions are identified by a mandatory *id attribute* and an optional *name attribute*. A reaction can be seen as a directed edge connecting a set of reactants to a set of products. The set of modifiers can be seen as an attribute of the edge. The reversibility attribute, when true, implicitly asserts that the role of reactants and products in the reaction can be reversed. A single species in an SBML document can participate in one or more reactions as reactant, product or modifier. SBML specifications allow the definition of species that do not take part in any reaction and reactions that have empty sets of reactants, products and modifiers.

Thus, the topology of an SBML map can be described by a *labeled multidigraph* (i.e. a directed graph that allows multiple edges between the same pair of nodes), where a single SBML reaction is spliced into a set of directed edges, connecting every single reactant with every single product in the reaction. The modifiers are treated as edges’ attributes and the compartments are treated as vertices’ attributes. In our model *incomplete* reactions (empty set of reactants or products) and *isolated* species are ignored.

### MIMO graph comparison procedure

The algorithmic procedure implemented in MIMO computes the overlap between two multidigraphs by detecting the largest possible *consistent* set of matches between shortest paths in the two graph structures. A matching is *consistent* if it induces a one-to-one mapping between subsets of reaction pathways, entities (species) and compartments. Two user-defined parameters are required in order to constrain the overlap: 1. *Maximum length N*: specifies the maximum-length for the computation of the shortest paths; 2. *List of allowed/forbidden matches between nodes and reaction pathways*: this list is used in step (ii) below to define the sets of *equivalences* between *species* and *reaction pathways*.

A short introductory description of the main points characterizing the algorithm is given below (see Additional file 1 for details). 

i) **Simple paths computation**. The algorithm computes and stores the set of *simple paths* of the two input graphs. A simple path is a *valid path* of *maximum lengthN* connecting two species. Moreover, a simple path is *non-overlapping*, i.e. it identifies a non-overlapping chain of SBML *reactions ids*. Finally, a simple path between two species is required to be *minimal*, i.e. all simple paths are shortest paths. In short, a simple path between two species is defined as the shortest chain of reactions connecting the two species. Given a pair of species, there can be distinct reaction chains of minimal length connecting the pair (by definition, a simple path is allowed to start and end at the same species).

ii) **Simple paths matching**. Two simple paths are considered *equivalent*, if they can be matched at the extremes, i.e. if they have equivalent initial and terminal species. By default, two species are considered equivalent if they have the same name attribute. Even when two paths are equivalent at the extremes, they are not matched if: a) the matching between the corresponding reaction pathways or species is explicitly forbidden by the user (user-defined parameters); b) there are matchable subpaths, i.e. if there are two equivalent intermediate species in the two paths; c) the matching does not induce a one-to-one mapping between species and compartments. For example, a simple path starting and ending at the same species (or respectively compartment) cannot be matched with a simple path connecting two distinct species (respectively compartments). The simple paths matching procedure naturally induces gaps and mismatches between two linear paths.

iii) **Maximal sets of compatible simple path pairs**. A subset of equivalent path pairs is *consistent* if it induces a one-to-one mapping between species, compartments and reaction pathways and it is *maximal* if it is not a proper subset of any other consistent set. This issue is recoded (in the standard way) as the computation of the *maximal clique* of the *compatibility graph* on the full set of equivalent path pairs, computed with the standard Bron-Kerbosh Version 2 (BKv2) algorithm [24].

In detail, the compatibility graph is an undirected graph (without self loops) that describes the compatibility between pairs of matched paths. By construction each vertex of the compatibility graph corresponds to one pair of matched paths and an edge between two vertices indicates that two pairs of paths are *compatible*. Two pairs of matched paths are compatible if the induced mapping between reaction pathways is one-to-one, i.e. a chain of reaction ids in one graph can be matched at most with a unique chain of reactions ids in the other graph, and, in particular, every single reaction id is required to belong at most to a unique path of reaction ids or to its reversed chain. Moreover, two pairs of matched paths are compatible if the induced mapping between vertices and compartments is one-to-one. A subset of equivalent path pairs respecting these rules is *consistent*, in the sense that it induces a one-to-one mapping between species, compartments and reaction id pathways.

The maximal clique detection is computationally intractable. In order to make this phase more efficient, the clique computation is iteratively performed in *N* steps, where, at each step *i*=1,…,*N* only the subset of path pairs of maximum length *i* is considered. This heuristic approach has the advantage of speeding up the computation, while giving more importance to the matches between the shortest biological pathways in the two maps. Intuitively, the procedure iteratively builds a solution by detecting first the *safest similarities* among the two graphs. After the computation of the maximal compatible subset, the procedure sets-up the mapping between the modifiers that appear in the selected pathways.

iv) **Overlap score**. A consistent subset *E* of equivalent path pairs detects a subgraph matching between the two input graphs, *G*_1_ and *G*_2_. The *comparison scoreS* associated to the subgraph matching *E* is defined as

(1) $$S(G_{1},E)=\frac{\underset{r\in R_{1}}{\sum }w(r)}{\left|R_{1}\right|},$$

where $R_{1}$ is the set of reaction identifiers in *G*_1_ and *w*(*r*) is a *weight* associated to the reaction *r*, computed as

(2) $$w(r)=\frac{\left|R_{E}(r)|+|P_{E}(r)|+|M_{E}(r)\right|}{\left|R(r)|+|P(r)|+|M(r)\right|},$$

where *R*(*r*),*P*(*r*),*M*(*r*) denote the set of Reactants, Products, Modifiers of reaction *r*, respectively, and *R*_*E*_(*r*),*P*_*E*_(*r*),*M*_*E*_(*r*) denote the set of Reactants, Products, Modifiers of reaction *r* contained in *E*, respectively. As a result *w*(*r*)=0 if *r* has no match in *G*_2_ and *w*(*r*)=1 if all reactants, products and modifiers of *r* have a match in *G*_2_ with respect to *E*. The score for *G*_2_ is computed equivalently by *S*(*G*_2_,*E*) (normalized on $\left|R_{2}\right|$). Note that, since the score *S* is normalized with respect to the size of the graph, it is in general not symmetric, i.e. in general *S*(*G*_1_,*E*)≠*S*(*G*_2_,*E*). This scoring function provides a value in [0,1] and roughly measures how much of the graph *G*_1_ is contained in *G*_2_ according to *E* (equivalently, for *G*_2_).

### Implementation details

Our software has been implemented in C language by using the libSBML [25] interface library version 4.1.0. The libSBML library provides routines for reading, writing, manipulating and validating SBML documents.

Our implementation takes as input two valid SBML documents (no conversion required into intermediate formats) and provides also the possibility to obtain as output, additionally to the comparison score, the computed overlap in SBML format. In particular, if requested by the user, the executable saves three SBML files related to the highest-scoring solution found. Two of these documents correspond to the SBML documents in which the non-matched reactions have been removed. In these files the matched reactions are saved in their full definition, i.e. all reactants, products and modifiers of the reaction are saved, even if they have not been matched in the computed overlap. The third SBML file contains the computed overlap between the two molecular interaction maps. In this file, every matched pair of reaction pathways is saved as a unique reaction. Only those reactants, products and modifiers that have been matched are saved into the document. In addition, in order to simplify the inspection of the output SBML documents, a text file listing the mappings between species, reactions and compartments is also saved.

The running time of our algorithm is bound by the running time of the BKv2 procedure [24], used in step (iii) for the computation of all maximal cliques of the compatibility graph. The BKv2 algorithm is efficient in practice [26] but, even with clever pivoting strategies, its running time depends also on the number of possible maximal cliques in a graph, which can be exponential in the number of nodes [27]. In order to provide some control on the running time of the algorithm in the most complex cases, the implementation allows the user to bound the running time by specifying a maximum number of solutions and/or a maximum amount of time for the execution of the BKv2 procedure. This feature can be useful to allow *fast* queries on large databases.

## Results and discussion

We present the performances of MIMO in detecting functional relationships among the 56 human pathways of the manually-curated Reactome [28] database version 39 [29] (full list in Additional file 2: Table S1). We performed a leave-one-out test on the whole ensemble of maps, where every single pathway is used as a query graph and it is compared against all the other pathways in the dataset; the detected top scoring pathways (if any) are considered as *related* to the query.

We compared the performances of MIMO with those of SAGA [20], which has been explicitly designed for efficient graph-database querying and which is, to date, the only publicly available tool closely related to our work. We converted the SBML documents into the SAGA graph format. We remark that in the SAGA graph format it is not possible to encode the information related to modifiers, compartments and direction of the reactions. In SAGA (like in MIMO) the isolated entities in the SBML maps are not encoded during format. We ran SAGA with the default parameters for the graph database creation (the maximum allowed length for the fragment index creation is equal to 3) with the exception of the D_MAX parameter, which is, by default, set to 3 and in our test has been set to 1. The reason is that, with the default value the algorithm returns almost no match. Additionally, we performed the query phase with SAGA by allowing 0.0 as percentage of non-gap nodes (by using the default parameter 0.8, the algorithm returns no match in all cases). For performance comparison, we ran MIMO with maximum path length equal to 3 (the same distance as SAGA) and with a maximum running time equal to 1 minute for the BKv2 procedure. No relevant improvement in terms of quality/size of the overlaps was observed when testing larger time-bounds for the BKv2 procedure (up to 1h). This is due to the fact that, for most of the complex cases (i.e. maps with very large overlaps), an upper bound of 1h for the BKv2 procedure is still not sufficient to explore the entire space of the possible solutions. Larger time-bounds are impractical, requiring weeks of computation.

To warrant the comparisons to be as informative as possible, we performed two different kinds of experiments on two disjoint subsets of the 56 pathways in Reactome. The first set consists of the 36 human pathways in Reactome that share a non-empty subset of reactions with at least one of the other maps in the dataset. Since reactions in Reactome SBML maps are uniquely identified by their *id attribute*, the *a priori* amount of overlap between two maps can be assessed by simply counting the fraction of common reactions (i.e. number of common reactions/total number of reactions). This set of 36 maps is used to assess the performances of MIMO and SAGA in detecting similarities between biological pathways (*validation* test, Table 1). The second set consists of the remaining 20 human pathways for which no trivial relationships can be inferred by using the reaction *id attribute*; that is to say that these pathways, if analyzed by the same overlap principle, share no common set of reactions with other maps in Reactome. Therefore such pathways are used to *infer* possible novel functional similarities between human pathways (*inference* test, Table 2). MIMO appears to be able to identify the largest of such non trivial solutions. A *validation* and an *inference* example are discussed in details in the final part of each section.

**Table 1:** Comparison of Reactome biological pathways

**Query**	**Reactome**			**MIMO**				**SAGA**		
	**Top-Hit(s)**	**Score**		**Top-Hit(s)**	**Score**	**AUC**		**Top-Hit(s)**	**Score**	**AUC**
R75829	R11061,R9417,	1.0000		R11061	0.8105	0.99		R11061,R9417,	117	0.88
	R16888,R111040,							R16888,R111040,		
	R9470							R9470		
R634	R9470,R9417,	1.0000		R9470,R9417,	0.9778	1.00		R9470,R9417,	66	1.00
	R498,R16888,			R498,R16888,				R498,R16888,		
	R111040,R11061,			R111040,R11061				R111040,R11061,		
	R6900							R6900		
R383	R152	1.0000		R152	1.0000	1.00		R152	291	1.00
R111064	R9470,R9417,	1.0000		R9470,R9417,	0.9778	0.96		*N/A*	-	-
	R16888,R11061			R16888,R11061						
R1675	R71	0.8254		R71	0.8087	1.00		R71	504	0.75
R16888	R11061,R9417	0.6875		R11061	0.6600	0.96		R11061,R9417,	399	0.80
	R9470							R9470		
R9417	R9470	0.5490		R9470	0.5451	0.95		R9470,R16888,	480	0.80
								R11061		
R1788	R71	0.5333		R71	0.5224	0.99		R71	409	0.75
R111040	R9470,R9417,	0.5068		R9470	0.5160	0.99		R9470, R9417,	360	1.00
	R16888,R11061							R16888,R11061		
R9470	R9417	0.5000		R11061	0.5196	0.96		R9417,R16888,	534	0.80
								R11061		
R13552	R604	0.4384		R604	0.4329	1.00		R604	431	0.75
R11061	R9470, R9417,	0.3313		R9470	0.3495	0.96		R9470, R9417,	819	0.80
	R16888							R16888		
R152	R383	0.3043		R383	0.3067	0.98		R383	870	1.00
R71	R1675	0.2989		R1675	0.2928	0.88		R17015	1275	0.85
R498	R11061	0.2985		R11061	0.4492	1.00		R11061,R6900	315	1.00
								R634,R16888	315	
								R111040,R9417	315	
								R9470	315	
R17015	R71	0.2115		R71	0.2008	1.00		R71	744	1.00
R111057	R6844	0.1667		R6844	0.2333	1.00		*N/A*	-	-
R1538	R152	0.1622		R152	0.3102	1.00		*N/A*	-	-
R474	R15518	0.1569		R15518	0.2220	0.91		R1505	354	0.60
R13433	R22258	0.1562		R22258	0.2132	0.97		R1698	782	0.54
R6844	R111057	0.1429		R111057	0.2333	1.00		*N/A*	-	-
R604	R13552	0.1100		R13552	0.1093	0.99		R13552	1659	0.55
R11123	R17015	0.1071		R17015	0.1454	1.00		R17015	342	1.00
R1505	R474	0.1067		R14797	0.2196	1.00		R474	366	0.56
R17044	R9470,R9417,	0.0909		R9470,R9417,	0.1803	0.94		*N/A*	-	-
	R16888,R11061,			R16888,R11061						
	R111064,R14797									
R14797	R11061	0.0700		R11061	0.0926	0.95		*N/A*	-	-
R13685	R14797	0.0682		R14797	0.0969	0.97		R15518	598	0.58
R15518	R474	0.0653		R474	0.0872	0.86		R13685	1067	0.57
R6900	R11061	0.0557		R11061	0.0805	0.98		R11061,R9417,	2909	0.91
								R16888,R9470		
R9431	R15518	0.0526		R13433	0.1637	0.94		R15518	112	1.00
R22258	R13433	0.0514		R13433	0.0744	1.00		R111083	1511	0.70
R111083	R474	0.0465		R13	0.2151	0.96		R22258	166	0.93
R13698	R15380	0.0385		R15380	0.3333	1.00		*N/A*	-	-
R299	R71	0.0345		R71	0.0345	1.00		*N/A*	-	-
R13	R474	0.0263		R22258	0.1303	0.97		R11193	688	0.79
R15380	R13698	0.0185		R1505	0.0260	0.98		*N/A*	-	-

Validation test. The Reactome score (between 0 and 1) denotes the fraction of reactions in the query map contained in the target map (see “Validation test” Section). MIMO’s score (between 0 and 1) has been defined in Step (iv) of MIMO’s procedure. SAGA’s score (integer ***≥0***) denotes the level of dissimilarity between two maps: the lower the score, the higher the similarity (N/A means no hit found). The AUC values have been computed by using as gold standard the Reactome reaction overlap scores: a Reactome score greater than 0 between two maps means *related* and a score equal to zero *not-related*.

**Table 2:** Comparison of Reactome biological pathways

**Query**	**MIMO**			**SAGA**	
	**Hits**	**Score**		**Hits**	**Score**
R11045	R152	0.2667		N/A	-
R11193	R22258	0.2246		R13433	293
R1698	R22258,R11193	0.1439		R13433	454
R12508	R22258	0.1208		N/A	-
R22172	R152	0.1203		N/A	-
R11044	R14797,R11061	0.1200		N/A	-
R21303	R6900	0.0952		N/A	-
R21257	R14797	0.0863		N/A	-
R6185	R71	0.0800		R71,R1788	667
R12034	R11045,R6844,R21303,R111057	0.0667		N/A	-
R111183	R71,R13685,R13433,R13	0.0571		N/A	-
R111155	R11061	0.0376		N/A	-
R216	R22258	0.0261		N/A	-
R578	R634,R498,R16888,R111040	0.0210		N/A	-
R6167	R15518	0.0187		N/A	-
R75925	N/A	-		N/A	-
R27161	N/A	-		N/A	-
R24941	N/A	-		N/A	-
R12529	N/A	-		N/A	-
R12472	N/A	-		N/A	-

Inference test. MIMO’s score (between 0 and 1) has been defined in Step (iv) of MIMO procedure. SAGA’s score (integer ***≥0***) denotes the level of dissimilarity between two maps: the lower the score, the higher the similarity (N/A means no hit found).

From a computational point of view, both MIMO and SAGA are extremely efficient (for the benchmark under test), with a time ratio between MIMO and SAGA of ∼4 when the necessary format conversion time (dataset upload into the postrgreSQL database) is included and ∼5 when excluded. This, however, comes at a cost in terms of the quality of the overlaps identified, including a number that is totally or partially missed by SAGA (Tables 1 and 2).

### Validation test

**Figure 1:**
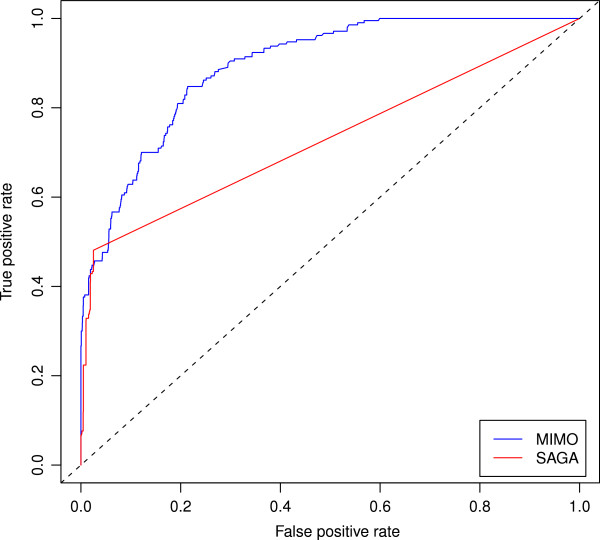
**ROC curve for the comparative performances of MIMO and SAGA over the 36 pathways in the validation test.** The test scores are the ones obtained from each algorithm and the gold standard is the overlap detected directly in Reactome: a Reactome score (see “Validation test” Section) greater than 0 between two maps means *related* and a score equal to zero *not-related*. SAGA ROC curve’s shape is due to the numerous missing overlaps. AUC MIMO = 0.89, AUC SAGA = 0.73.

The results of the biological validation test are summarized in Table 1. For visualization purposes, we show only the top-scoring hit for each query pathway. Multiple hits are shown only when the comparison score is exactly the same. The full list of comparisons is available as supplementary data (see Additional file 3: Table S2). Overlaps for which SAGA does not return any hits (9 out of 36), are due to: (i) the quality filtering used (a match is not returned if it has *P*-value ≤0.01) and (ii) the fragment procedure which forbids the match between *linear pathways* in two graphs. As a consequence, non-trivial overlaps (i.e. >16*%* common reactions) such as the ones related to the pairs (R111064, R9470), (R111057, R6844) and (R1538, R152) are lost (see Table 1). In general, from a qualitative point of view SAGA always identifies overlaps smaller than the ones identified by MIMO. One notable example is the query R383 which is a subgraph of map R152 (correctly identified by MIMO) and for which SAGA is able to detect a mapping for only 12 out of 109 nodes. From a quantitative point of view, MIMO is able to correctly detect the best hits for 29 out of 36 queries against 20 out of 36 identified by SAGA. Moreover, when -due to the time-bound constraints imposed to the algorithm- the optimal solution is not the top ranking, MIMO always identifies it in the top four positions (see Additional file 3: Table S2). Finally, performance comparison in terms of Areas Under the ROC curves (AUC) for each single query (Table 1) and on the entire set of 36 maps (Figure 1) confirms that the classification performance of MIMO is superior to that of SAGA.

When querying map R111083 (see Table 1) MIMO detects a non-trivial overlap with R13 (scoring hit 0.2151 in Table 1). For MIMO, the similarity of R111083 with R13 is higher than with R474 (as expected by inspecting Reactome’s hit, see Table 1). Interestingly, this overlap detects a non-trivial relationship between R111083 and R13 which cannot be directly inferred from Reactome, as in fact, such maps have no common reactions (see Additional file 3: Table S2).

#### The citric acid (TCA) cycle and respiratory electron transport *(*R111083) vs Metabolism of amino acids and derivatives *(R13)*

**Figure 2:**
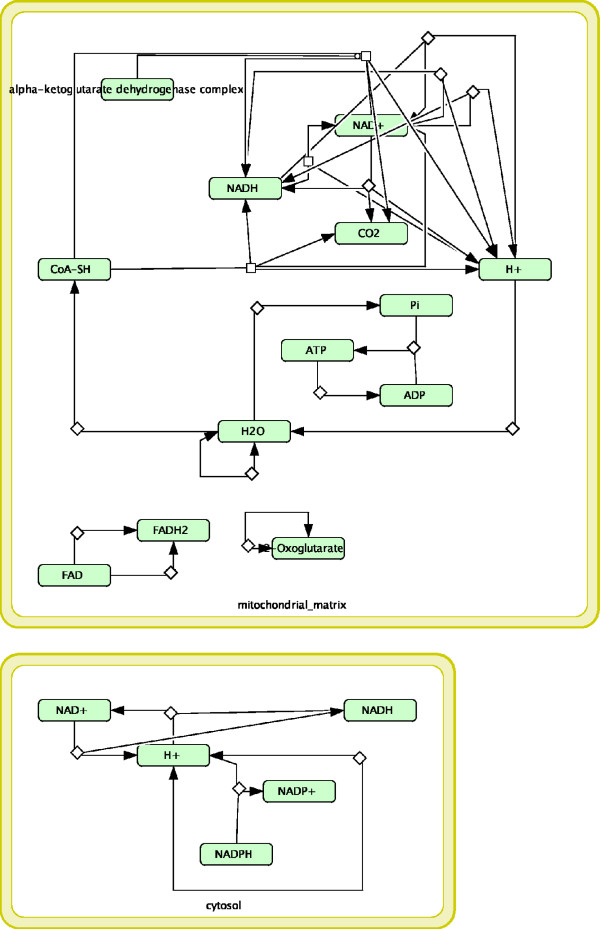
**R111083 vs R13.** The citric acid (TCA) cycle and respiratory electron transport (R111083) vs Metabolism of amino acids and derivatives (R13).

The matched subgraph of these two pathways is shown in Figure 2. The overlap involves entities located into the “mitochondrial matrix” and “cytosol” compartments. The most interesting biological relationship regards the set of matched reactions in the “mitochondrial matrix” and the role of the “alpha-ketoglutarate dehydrogenase complex”, which is an enzyme complex involved in lysine degradation and tryptophan metabolism.

The junction between the two original maps in the “mitochondrial matrix” derived from a chain of events that link the two reactions. The citric acid cycle (in R111083) comprises a series of enzyme-catalysed chemical reactions of central importance in all living cells that use oxygen as part of cellular respiration. In eukaryotic cells, the citric acid cycle occurs in the matrix of the mitochondrion [30] and starts with acetyl-CoA. In particular, pyruvate is derived primarily from glucose 6-phosphate, alanine, and lactate and is converted into acetyl-CoA, the main input for the citric acid cycle. In a separate process, protein catabolism (part of proteins metabolism, R13), proteins are broken down by proteases into their constituent amino acids, in a complex process due to the large number of enzymes and metabolites involved. Following the metabolic fate of carbon atoms in the amino acids, it is possible to trace all the major metabolic intermediates because of the close interaction of amino acid metabolism with the citric acid cycle. In fact, carbon backbones of amino acids become a source of energy once they are converted to acetyl-CoA, either directly (Ketogenic amino acids) or indirectly via degradation to pyruvate (Glucogenic amino acids) and enter into the citric acid cycle [31]. Acetyl-CoA is then the junction between the citric acid cycle and the metabolism of amino acids. As it is visible from the overlap of these two pathways, most of the species and reactions are located in the mitochondrion and the main reaction: alpha-ketoglutarate dehydrogenase complex + NAD + CoA + Succinyl CoA + CO2 + NADH was successfully identified by MIMO. Clearly, the overlap between these two pathways shows how amino acids enter the citric acid cycle and follow the different steps of the citric acid cycle. Interestingly, this could not be inferred in SAGA, the reason being that two very important species, alpha-ketoglutarate dehydrogenase complex and CoA, were not highlighted in this overlap (data not shown).

### Inference test

The results of the biological inference test are summarized in Table 2. For visualization purposes, as for Table 1, only the top-scoring hit for each query pathway are shown. The full list of comparisons is available as supplementary data (see Additional file 3: Table S2).

From Table 2, SAGA detects some similarities only for three out of 20 queries in the dataset, compared to 15 of MIMO. This suggests that MIMO is more sensitive than SAGA in detecting similarities among biological graphs. However, most of such overlaps, though effective from a computational point of view, do not highlight functional markers, but are, in most of the cases, confined to very common molecules (H+, H20, Oxygen, NAD+, NADH). The impact of such common molecules in the *computational* analyses of biological networks can represent indeed an issue as it distorts the evaluation of topological network parameters such as *centrality* or *pathway length*[32]. However, the *biological* interest of such overlaps may not be discarded *a priori*, and since non-interesting overlaps can be easily detected by visual inspection of the outputs we here chose to maintain them (like tha authors of SAGA did), and give the end-user the option to keep or remove them. Nevertheless, in this inference test at least in one case, namely for maps R11045 and R152, MIMO identifies a non-trivial overlap, which is not identified by SAGA (see Table 2). This example is discussed in detail below.

#### Signaling by Wnt *(R11045)* vs Cell Cycle, Mitotic *(R152)*

**Figure 3:**
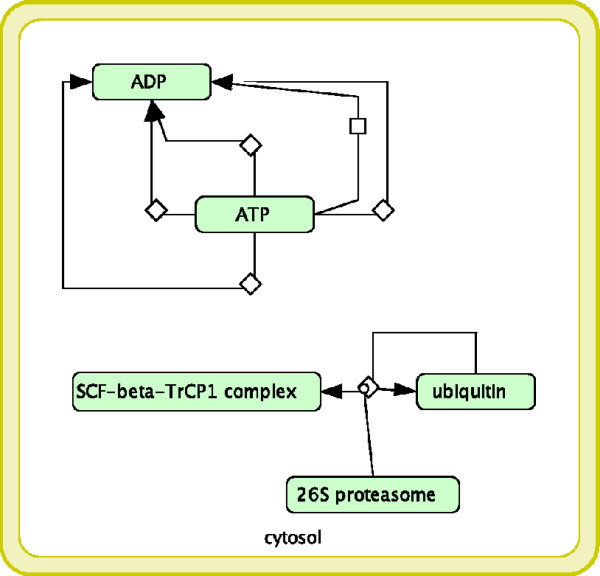
**R11045 vs R152.** Signaling by Wnt (R11045) vs Cell Cycle, Mitotic (R152).

The matched subgraph of these two pathways is shown in Figure 3. The overlap involves entities located into the “cytosol” and it is related to the role of the “SCF- *β*-TrCP1 complex” and the “26S proteasome”.

The Wnt signaling pathway (R11045) is critically involved in the early development of complex, multi-cellular organisms controlling early axis formation, limb patterning and organogenesis [33]. Abnormal Wnt signaling is often related to severe human diseases, including cancer, osteoporosis and other degenerative disorders [34]. The replication of the genome and the subsequent segregation of chromosomes into daughter cells (R152) are controlled by a series of events collectively known as the cell cycle. In particular, a family of protein (serine/threonine kinases) known as the cyclin-dependent kinases (CDKs) controls the progression through the cell cycle [35]. Recent work has implicated Wnt components in regulating mitotic events, suggesting that cell cycle and Wnt signaling are directly linked [36]. Interestingly, MIMO can highlight this connection and additionally it can also provide details regarding the molecules involved. In fact, by reading the output file, it is possible to argue that this happens via the activity of SCF- *β*-TrCP1 complex (an ubiquitin ligase) whose substrates can be divided into two main groups: direct regulators of CDKs, regulators of gene transcription or both. *β*-catenin is the substrate of SCF- *β*-TrCP1 in the Wnt signaling pathway [37] where it plays a key role. In particular, phosphorylated *β*-catenin is recognized and ubiquitinated by the SCF- *β* TrCP ubiquitin ligase complex and is subsequently degraded by the proteasome [38]. Similarly, some of the CDKs, such as cdc20, are also the substrate of SCF- *β* TrCP in the cell cycle. From there, once they have been identified by SCF- *β* TrCP, all substrates enter the process of proteasomal degradation, the identified common process.

## Conclusions

MIMO (Molecular Interaction Map Overlap) is a tool for biological graph matching. The main features of MIMO are: (i) *Easy-to-use*: MIMO takes as input biological networks encoded with the Systems Biology Markup Language (SBML) standard. The SBML standard is widely adopted for biological network modeling and is flexible enough to allow the encoding of quite complex molecular interactions. Most importantly, the choice to adopt a standard format as input avoids the pre-processing phase needed to convert molecular interaction maps in a non-standard format removing all the consequent burden. (ii) *Flexibility*: MIMO implements a flexible procedure for sub-graph matching, which naturally allows the introduction of gaps and mismatches and permits (if required) supervised queries incorporating *a priori* biological information. (iii) *Computational efficiency*: while the subgraph matching problem is computationally intractable, MIMO implementation is fast enough to allow multiple queries on graph databases.

The capabilities of MIMO have been highlighted by performing a one-to-one comparison on all human pathways in the Reactome database. The experimental tests prove that MIMO is flexible and efficient enough to make it a suitable tool for biological pathway comparisons.

## Availability and requirements

**Project Name**: Molecular Interaction Map Overlap;**Project home page**: http://www.picb.ac.cn/ClinicalGeno micNTW/software.htm;**Operating system(s):** Linux, Mac Os X;**Programming language:** C;**Other requirements:** libSBML;**Licence:** GPL3;**Any restrictions to use by non-academics:** No.

## Competing interests

The authors declare that they have no competing interests.

## Authors’ contributions

PDL and CN designed the project and wrote the manuscript. PDL designed and implemented the algorithmic procedure, and carried out all computational experiments. GW interpreted the biological results. All the authors read and approved the final manuscript.

## Supplementary Material

Additional file 1 Detailed description of the algorithmic procedure. Click here for file

Additional file 2: Table S1 List of Reactome’s biological pathways in human. Click here for file

Additional file 3: Table S2 Full list of comparison scores. Click here for file
