# Supplementary material for: MIMO: an efficient tool for molecular interaction maps overlap
Source: BMC Bioinformatics. 2013 May 15;14:159. doi: 10.1186/1471-2105-14-159 (PMC3680968; doi:10.1186/1471-2105-14-159)
Supplement: Additional file 1 — Detailed description of the algorithmic procedure. [file 1471-2105-14-159-S1.pdf]

MIMO Details

1 SBML format and graph model

The Systems Biology Markup Language (SBML) [1] is a free XML-based format for representing molecular interaction networks. In the following, we only describe those components of SBML documents that are used in our comparison algorithm. For a detailed description of the latest SBML release refer to [2].

An SBML document specifies a set of entities, generally denoted with the term *species*, that take part in *reactions*. An SBML specie has two mandatory attributes: the *id attribute*, used to uniquely identify the species entities in the document and the *compartment* attribute, a type of location where the reacting entities are located. A specie has also an optional *name attribute*, of type string. A *reaction* component is a statement that links one or more species. A reaction is defined in terms of the participating species and it consists of:

- a possibly empty set of species called *reactants*
- a possibly empty set of species called *products*
- a possibly empty set of species called *modifiers*
- reversibility attribute: true/false

The set of reactants, products and modifiers does not uniquely identify a reaction: as for species, every reaction is identified by a mandatory *id attribute* and an optional *name* attribute. A reaction can be seen as a directed edge connecting a set of reactants to a set of products. The set of modifiers can be seen as an attribute of the edge. The reversibility attribute, when equal to true, implicitly asserts that the role of reactants and products in the reaction can be reversed. A specie in the SBML document can participate in one or more reactions as reactant, product or modifier. SBML specifications allow the definition of species that do not take part in any reaction and reactions that have empty sets of reactants, products and modifiers.

The topology of an SBML map can be described by a *labeled multidigraph*, i.e. a directed graph that allows multiple (parallel) edges between the same nodes. In detail, given an SBML document D, denote with $\mathcal{S}, \mathcal{R}, \mathcal{N}$ and $\mathcal{C}$ the set of *SBML specie id attributes*, *SBML reaction id attributes*, *SBML specie name attributes* and *SBML compartment name attribute*, respectively. The multidigraph G related to D is a 8-tuple $(V, E, s, t, \gamma, \kappa, \lambda, \mu)$ where

- $V \subseteq \mathcal{S}$ is a set of *vertices*. Each node in V represents a specie in the SBML document D. In our model, the set V is a subset of all species in D since it contains only those species in the SBML document that have a non-empty name attribute and that are involved in at least one reaction for which both the set of reactants and modifiers are nonempty.
- E is a set of *edges*. There is an edge between every pair of nodes $v, v' \in V$ such that v is the reactant and v' the product of a reaction in D or v is the product and v' the reactant of a *reversible* reaction in D. Since two species v, v' can be both involved in different reactions, there can be several different edges starting from v and ending in v' (and conversely).
- $s : E \to V$ and $t : E \to V$ are two functions that map every edge $e \in E$ to its *starting vertex* $s(e) \in V$ and its *terminating vertex* $t(e) \in V$.
- $\gamma : V \to \mathcal{N}$ is a labeling function that maps every vertex in V to the *name attribute* of the corresponding specie.

- $\kappa : V \to \mathcal{C}$ is a labeling function that maps every vertex in V to the *compartment name attribute* of the corresponding specie.
- $\lambda : E \to \mathcal{R}$ is a labeling function that maps every edge in E to the *id attribute* of the corresponding reaction. Note that the λ function is not one-to-one, since multiple edges can have the same id attribute.
- $\mu : E \to 2^V$, where 2^V denotes the set of all subsets of V (including the empty set), is a labeling function that maps every edge $e \in E$ to the *set of modifiers* of the reaction $\lambda(e)$.

We do not encode in our multidigraph those species that are *isolated* in the model and those that have no name attribute. Moreover we do not encode those reactions that have an empty set of reactants or products while the set of modifiers (defined as an attribute of the edges) is allowed to be empty.

In order to simplify the notation, we will abuse the above notation in the following way. The functions s, t, γ, λ and μ will be ambiguously used on distinct graphs. For instance, we will always denote with s the function that returns the starting vertex of an edge, even if s is used on edges belonging to different graphs (in which case, the domain of s is different on the two graphs). Moreover, we assume that these functions are naturally extended to *paths* in the following way. Given $p = e_1...e_n \in E^+$, we use the following conventions:

- $s(p)$ denotes $s(e_1)$ and $t(p)$ denotes $t(e_n)$,
- $\lambda(p)$ denotes the sequence $\lambda(e_1)..\lambda(e_n)$,
- $\mu(p)$ denotes the set $\cup_{i=1}^{n} \mu(e_i)$.

2 Graph comparison procedure

In our model, the overlap between two multidigraphs $G_1 = (V_1, E_1, s, t, \gamma, \kappa, \lambda, \mu)$ and $G_2 = (V_2, E_2, s, t, \gamma, \kappa, \lambda, \mu)$ is obtained by detecting the largest possible (consistent) set of compatible matchings between non-overlapping shortest paths in the two graphs. In particular, this induces a one-to-one mapping between subset of reaction pathways, entities (species) and compartments. This approach is quite powerful and flexible and allows gap and mismatches identification *a posteriori* from the computed solution.

The implementation of our algorithm relies on two user-defined parameters:

1. *Maximum length of simple pathways*: N specifies the maximum-length for the computation of the shortest paths.
2. *List of allowed/forbidden matchings between nodes and reaction pathways*: the list is used to constraint or extend the set of possible mappings between the two graphs. In detail, given two species $v_1 \in V_1$ and $v_2 \in V_2$, the list can contain mappings of the form:
   - $v_1 \leftrightarrow v_2$, i.e. the specie v_1 can be mapped with specie v_2 even if $\gamma(v_1) \neq \gamma(v_2)$ (v_1 and v_2 have different name attributes)
   - $v_1 \not\leftrightarrow v_2$, i.e. the specie v_1 cannot be mapped with specie v_2 even if $\gamma(v_1) = \gamma(v_2)$ (v_1 and v_2 have same name attributes)

   Thus we say that two species $v_1 \in V_1$ and $v_2 \in V_2$ are *equivalent*, $v_1 \sim v_2$, if:

   (a) $\gamma(v_1) = \gamma(v_2)$ (i.e. v_1 and v_2 have the same name attribute) and the mapping $v_1 \not\leftrightarrow v_2$ is not explicitly forbidden by the user or

(b) $\gamma(v_1) \neq \gamma(v_2)$ but the mapping $v_1 \leftrightarrow v_2$ is explicitly allowed by the user.

Furthermore, given two pathways p_1 in G_1 and p_2 in G_2, with $\lambda(p_1) = r_1..r_m$ and $\lambda(p_2) = s_1..s_n$, the user is allowed to explicitly forbidden the mapping between p_1 and p_2, $r_1..r_m \not\leftrightarrow s_1..s_n$.

The algorithmic procedure implemented in MIMO consists of 4 main steps.

i) Simple paths computation. Given two graphs G_1 and G_2, the algorithm computes and stores the set of *simple paths*, P_1 and P_2, of G_1 and G_2, respectively. Formally, given a graph $G = (V, E, s, t, \gamma, \kappa, \lambda, \mu)$, a sequence of edges $p = e_1...e_n \in E^+$ is a *simple path* if:

(p1) p is a *valid path* in G: $\forall 1 \leq i < n, t(e_i) = s(e_{i+1})$

(p2) p is *non-overlapping*: $\forall 1 \leq i \neq j \leq n, \lambda(e_i) \neq \lambda(e_j)$

(p3) p is *minimal*: $\nexists p' \in E^+$, with properties (p1-2), such that $|p| > |p'|$ and $s(p) = s(p'), t(p) = t(p')$

(p4) p is of *maximum length* N.

A simple path is a *valid path* in G (p1) of *maximum length* N (p4). Moreover, a simple path is *non-overlapping* in G in the sense that it identifies a non-overlapping chain of reactions ids (p2). Finally, a simple path between nodes v, v' is required to be *minimal* (p3), i.e. all simple paths are shortest paths. Note that, by definition, a simple path is allowed to start and end at the same vertex.

In conclusion, this step returns two lists of simple paths P_1 and P_2 of maximum length N for G_1 and G_2, respectively.

ii) Simple paths matching. In this phase, the algorithm computes and stores the list of *equivalent* simple paths in the two maps. The path matching procedure can be formalized in the following way. Consider two simple paths $p_1 = e_1...e_m \in P_1$ and $p_2 = e'_1...e'_n \in P_2$. Then p_1, p_2 are *equivalent* if the following four conditions hold:

(m1) p_1 and p_2 have *equivalent extremes*: $s(p_1) \sim s(p_2)$ and $t(p_1) \sim t(p_2)$

(m2) p_1 and p_2 *do not contain matchable subpaths*: $\forall i \in [1, m), j \in [1, n), t(e_i) \not\sim t(e'_j)$

(m3) p_1 and p_2 identify a *one-to-one mapping between species and compartments*:

- $s(p_1) = t(p_1)$ if and only if $s(p_2) = t(p_2)$
- $\kappa(s(p_1)) = \kappa(t(p_1))$ if and only if $\kappa(s(p_2)) = \kappa(t(p_2))$

(m4) the mapping $\lambda(p_1) \not\leftrightarrow \lambda(p_2)$ is not explicitly forbidden by the user

In order to be equivalent, two simple paths are required to have equivalent initial and terminal vertices (m1). The matching between two simple paths is not considered if the matching between the corresponding reaction pathways is explicitly forbidden by the user (m4), if they do not induce a one-to-one mapping between species and compartments (m3) and if they contain matchable subpaths (m2), i.e. if there are two equivalent intermediate nodes in the two paths.

In conclusion, this step returns a set of equivalent path pairs

$$E = \{(p_1, p_2) \in P_1 \times P_2 \mid p_1 \in P1 \text{ and } p_2 \in P2 \text{ are equivalent simple paths according to rules (m1-4)}\}.$$

iii) Maximal sets of compatible simple path pairs. In this phase, the procedure selects the *maximal consistent* subset of E, computed at the previous step (ii). Note that each pair of paths in E identifies a small overlap between G_1 and $G2$. Not all of such small overlaps are *compatible/consistent.*A subset of E is *consistent* if it induces a one-to-one mapping between species, compartments and reaction pathways and it is *maximal* if it is not a proper subset of any other consistent set. The problem of computing the maximal consistent subset of E can be recoded as the problem of computing the *maximal clique* of the *compatibility graph* on E. The *maximal clique* of the compatibility graph is computed here with the standard Bron-Kerbosh Version 2 (BKv2) algorithm [3]. In the rest of this section we describe in detail the exact procedure used to select a maximal consistent subset of E.

The compatibility graph is an undirected graph (without self loops) that describes the compatibility between pairs of matched paths. Each vertex of the compatibility graph corresponds to one pair of matched paths in E. An edge between two vertices of the compatibility graph is present if and only if the two pairs of matched paths respect the following two properties. Two pairs of equivalent paths $(p_1, p_2), (p'_1, p'_2) \in E$ are *compatible* if:

(c1) *the induced mapping between reaction pathways is one-to-one*: there is a common reaction between $\lambda(p_1) = r_1..r_m$ and $\lambda(p'_1) = r'_1..r'_{m'}$ or between $\lambda(p_2) = s_1..s_n$ and $\lambda(p'_2) = s'_1..s'_{n'}$, i.e.

$$\{r_1, .., r_m\} \cap \{r'_1, .., r'_{m'}\} \neq \emptyset \text{ or } \{s_1, .., s_n\} \cap \{s'_1, .., s'_{n'}\} \neq \emptyset$$

if and only if

- $\lambda(p_1) = \lambda(p'_1)$ and $\lambda(p_2) = \lambda(p'_2)$, or
- $\lambda(p_1)$ and $\lambda(p_2)$ are the reverse of $\lambda(p'_1)$ and $\lambda(p'_2)$, respectively, i.e. $\lambda(p_1) = r_1..r_m = r'_{m'}..r'_1$ and $\lambda(p_2) = s_1..s_n = s'_{n'}..s'_1$

(c2) *the induced mapping between vertices and compartments is one-to-one*: consider the set of vertices mappings

$$X = \{s(p_1) \leftrightarrow s(p_2), t(p_1) \leftrightarrow t(p_2), s(p'_1) \leftrightarrow s(p'_2), t(p'_1) \leftrightarrow t(p'_2)\}$$

Then $\forall x \leftrightarrow y, x' \leftrightarrow y' \in X$, it must be

- $x = x'$ if and only if $y = y'$
- $\kappa(x) = \kappa(x')$ if and only if $\kappa(y) = \kappa(y')$

Two pairs of matched paths are compatible if the induced mapping between reaction pathways is one-to-one, i.e. a chain of reaction ids in one graph can be matched at most with a unique chain of reactions id in the other graph, and, in particular, every single reaction id is required to belong at most to a unique pathway of reaction ids or its reverse (c1). Moreover, two pairs of matched paths are compatible if the induced mapping between vertices and compartments is one-to-one (c2). This last condition avoids, for example, a reaction starting and ending in one single compartment to be mapped with a reaction that spreads among two different compartments. Note that, a subset $E' \subseteq E$ for which each pair of matched paths respect rules (c1-2) assures that the subset E' is consistent in the sense that it induces a one-to-one mapping between species, compartments and reaction id pathways.

The computation of the maximal compatible subset of E is in general computationally hard both in terms of time and memory usage. For this reason, the computation of the maximal compatible subset of E is iteratively performed in N steps. First of all, E is spliced into N disjoint subsets, $E_1, ..., E_N$ where for $1 \leq i \leq N$

$$E_i = \{(p_1, p_2) \in E \mid |p_1| \leq i, |p_2| \leq i \text{ and } (|p_1| = i \text{ or } |p_2| = i)\},$$

where $|p|$ is the length of pathway p. Thus, at step 1, the procedure computes the compatibility graph on E_1 and a maximal clique on such compatibility graph with the BKv2 algorithm. The maximal clique on E_1 identifies an initial solution $E' \subseteq E_1 \subseteq E$. At step 2, the procedure selects from E_2 the subset $E'_2 \subseteq E_2$ of all path pairs that are compatible with all path pairs in E' with respect to rules (c1-2). As in the previous iteration, the procedure builds the compatibility graph on E'_2 and detects the maximal clique of such compatibility graph with the BKv2 procedure. The solution found is added to E'. This procedure is iteratively performed for N steps. At the end such iterative procedure, the set $E' \subseteq E$ is a maximal consistent subset of E and it encodes a solution for the overlap between G_1 and G_2. We remark that, due to our iterative procedure for the selection of the elements, while E' is always maximal, its is not necessarily the largest possible; that is, there can be maximal consistent subsets of E with a larger number of elements than E'. On the other end, our heuristic approach for the selection of E' has the advantage of speeding up the computation and of giving more importance to the shortest pathways matches first: in this sense, the procedure iteratively builds a solution by detecting first the most *safe* similarities among the two graphs,

When matching two paths, the matching between the modifiers (if present) computed directly is not convenient from a computational point of view therefore MIMO detects mappings between modifiers a posteriori. After the computation of the subset $E' \subset E$, the procedure setups the mapping between those modifiers that appear in the selected pathways. The procedure builds a list of modifier pairs (i.e. modifiers that can be mapped according to $\sim$), it builds the compatibility graph on such list and then detects a compatible set of mappings between modifiers by selecting a maximal clique on such compatibility graph.

iv) Overlap score. The maximal subset $E' \subseteq E$ computed in the previous step detects a subgraph matching between G_1 and G_2. The *comparison score* S associated to E' is defined as

$$S(G_1, E') = \frac{\sum_{r \in \mathcal{R}_1} w(r)}{|\mathcal{R}_1|},$$

where $\mathcal{R}_1$ is the set of reaction identifiers in G_1 and $w(r)$ is a *weight* associated to the reaction r, computed as

$$w(r) = \frac{|R_E(r)| + |P_E(r)| + |M_E(r)|}{|R(r)| + |P(r)| + |M(r)|},$$

where $R(r), P(r), M(r)$ denote the set of Reactants, Products, Modifiers of reaction r, respectively, and $R_E(r), P_E(r), M_E(r)$ denote the set of Reactants, Products, Modifiers of reaction r contained in E, respectively. As a result $w(r) = 0$ if r has no match in G_2 and $w(r) = 1$ if all reactants, products and modifiers of r have a match in G_2 with respect to E. The score for G_2 is computed equivalently by $S(G_2, E)$ (normalized on $|\mathcal{R}_2|$). Note that, since the score S is normalized with respect to the size of the graph, it is in general not symmetric, i.e. in general $S(G_1, E) \neq S(G_2, E)$. This scoring function provides a value in $[0, 1]$ and roughly measures how much of the graph G_1 is contained in G_2 according to E (equivalently, for G_2).

Implementation details

Our software has been implemented in C language by using the libSBML [4] interface library version 4.1.0. The libSBML library provides routines for reading, writing, manipulating and validating SBML documents. Our implementation takes in input two valid SBML documents and there is no need of conversion into an intermediate format.

The memory usage and the running time of our algorithm is generally bound by the running time of the BKv2 algorithm [3], used in step (iii) for the computation of all maximal cliques of the compatibility graph. The BKv2 algorithm is efficient in practice [5] but, even with clever pivoting strategies, its running time depends also on the number of possible maximal cliques in a graph, which can be exponential on

the number of nodes [6]. In order to provide some control on the running time of the algorithm in the most complex cases, we allow the user to bound the running time of the BKv2 procedure by specifying a maximum number of solutions and/or a maximum amount of time for the execution of the BKv2 procedure.

Finally, if required by the user, during the computation, the algorithm can avoid to take into consideration the *compartment constraints* (i.e. it does not assure that the mapping between compartments is one-to-one) and it can avoid to find a mapping between modifiers (so the reaction weight in the final score is normalized with respect to reactants and products only).

Our implementation provides also the computed overlap in SBML format. In particular, if requested by the user, the executable saves three SBML files related to the solution found. Two of these documents correspond to the SBML documents in which the non-matched reactions have been removed. In these files the matched reactions are saved in their full definition, i.e. all reactants, products and modifiers of the reaction are saved, even if they have not been matched in the computed overlap. The third SBML file contains the true overlap between the two molecular interaction maps. In such file, every matched pair of reaction pathways is saved as a unique reaction. Only those reactants, products and modifiers that have been matched are saved into the document. In addition, in order to simplify the inspection of such SBML documents, a text file listing the mappings between species, reactions and compartments is also saved.

References

1. Hucka M, Finney A, et al.: **The systems biology markup language (SBML): a medium for representation and exchange of biochemical network models**. *Bioinformatics* 2003, **19**(4):524–531.

2. Hucka M, Bergmann F, et al.: **The systems biology markup language (SBML): Language specification for level 3 version 1 core (release 1 candidate)**. *Nature Precedings* 2010.

3. Bron C, Kerbosch J: **Algorithm 457: finding all cliques of an undirected graph**. *Commun. ACM* 1973, **16**(9):575–577.

4. Bornstein BJ, Keating SM, Jouraku A, Hucka M: **LibSBML: an API Library for SBML**. *Bioinformatics* 2008, **24**(6):880–881.

5. Tomita E, Tanaka A, Takahashi H: **The worst-case time complexity for generating all maximal cliques and computational experiments**. *Theoretical Computer Science* 2006, **363**:28 – 42.

6. Moon J, Moser L: **On cliques in graphs**. *Israel Journal of Mathematics* 1965, **3**:23–28.
