# Supplementary material for: MIMO: an efficient tool for molecular interaction maps overlap
Source: BMC Bioinformatics. 2013 May 15;14:159. doi: 10.1186/1471-2105-14-159 (PMC3680968; doi:10.1186/1471-2105-14-159)
Supplement: Additional file 2: Table S1 — List of Reactome’s biological pathways in human. [file 1471-2105-14-159-S2.pdf]

Table 1. Reactome biological pathways for functional comparison

Id	Pathway Name
R75925	Amyloids
R578	Apoptosis
R1538	Cell Cycle Checkpoints
R152	Cell Cycle Mitotic
R111155	Cell-Cell communication
R22172	Chromosome Maintenance
R24941	Circadian Clock
R18266	Axon Guidance
R21303	Myogenesis
R13698	Regulation of beta-cell development
R111057	Signaling by NODAL
R27161	Transcriptional Regulation of White Adipocyte Differentiation
R15380	Diabetes pathways
R216	DNA Repair
R383	DNA Replication
R71	Gene Expression
R604	Hemostasis
R6185	HIV Infection
R6900	Immune System
R6167	Influenza Infection
R111183	Meiosis
R11123	Membrane Trafficking
R12508	Metabolism of nitric oxide
R1698	Metabolism of nucleotides
R111083	The citric acid (TCA) cycle and respiratory electron transport
R21257	Metabolism of RNA
R11193	Metabolism of vitamins and cofactors
R13	Metabolism of amino acids and derivatives
R1505	Integration of energy metabolism
R9431	Metabolism of porphyrins
R13433	Biological oxidations
R474	Metabolism of carbohydrates
R22258	Metabolism of lipids and lipoproteins
R17015	Metabolism of proteins
R1675	mRNA Processing
R17044	Muscle contraction
R13685	Neuronal System
R12472	Regulatory RNA pathways
R9417	Signaling by EGFR
R9470	Signaling by FGFR
R498	Signaling by Insulin receptor
R11061	Signalling by NGF
R16888	Signaling by PDGF
R12529	Signaling by VEGF
R111040	Signaling by SCF-KIT
R111064	DAG and IP3 signaling
R75829	PIP3 activatesAKT signaling
R634	RAF-MAP kinase cascade
R11044	Signaling by Rho GTPases
R12034	Signaling by BMP
R6844	Signaling by TGF beta
R299	Signaling by Notch
R14797	Signaling by GPCR
R11045	Signaling by Wnt
R13552	Integrin cell surface interactions
R1788	Transcription
R15518	Transmembrane transport of small molecules

The 56 Reactome biological pathways included in our functional comparison analysis (Version 39, Dec 14, 2011). Full names (**Pathway Name**) were abbreviated into Reactome identifier notation (**Id**).
